# Short and mid‐term characteristics of COVID‐19 disease course in athletes: A high‐volume, single‐center study

**DOI:** 10.1111/sms.14265

**Published:** 2022-11-20

**Authors:** Vencel Juhász, Liliána Szabó, Attila Pavlik, András Tállay, Dorottya Balla, Orsolya Kiss, Máté Babity, Nóra Sydó, Emese Csulak, András Benczúr, Anna Országh, Zsófia Gregor, Dávid Becker, Béla Merkely, Hajnalka Vágó

**Affiliations:** ^1^ Heart and Vascular Center Semmelweis University Budapest Hungary; ^2^ Department of Sports Medicine Semmelweis University Budapest Hungary; ^3^ Institute for Computer Science and Control Budapest Hungary

**Keywords:** athletes, COVID‐19, long COVID, post‐acute COVID syndrome, return to play, SARS‐CoV‐2 infection

## Abstract

**Introduction:**

At the pandemic's beginning, significant concern has risen about the prevalence of myocardial involvement after SARS‐CoV‐2 infection. We assessed the cardiovascular burden of SARS‐CoV‐2 in a large cohort of athletes and identified factors that might affect the disease course. We included 633 athletes in our study on whom we performed extensive cardiology examinations after recovering from SARS‐CoV‐2 infection. More than half of the athletes (*n* = 322) returned for a follow‐up examination median of 107 days after the commencement of their infection.

**Results:**

Troponin T positivity was as low as 1.4% of the athletes, where the subsequently performed examinations did not show definitive, ongoing myocardial injury. Altogether, 31% of the athletes' rapid training rebuild was hindered by persistent or reoccurring symptoms. Female athletes reported a higher prevalence of return to play (RTP) symptoms than their male counterparts (34% vs. 19%, *p* = 0.005). The development of long COVID symptoms was independently predicted by increasing age and acute symptoms' severity in a multiple regression model (AUC 0.75, CI 0.685–0.801). Athletes presenting with either or both cough and ferritin levels higher than >150 μg/L had a 4.1x (CI 1.78–9.6, *p* = 0.001) higher odds ratio of developing persistent symptoms.

**Conclusion:**

While SARS‐CoV‐2 rarely affects the myocardium in athletes, about one in three of them experience symptoms beyond the acute phase. Identifying those athletes with a predisposition to developing long‐standing symptoms may aid clinicians and trainers in finding the optimal return‐to‐play timing and training load rebuild pace.

## 1 INTRODUCTION

The COVID‐19 pandemic has had a significant impact on the athletes' world in several dimensions since its beginning in 2019. The pandemic has been globally present, with regularly emerging waves as new virus variants appear.^1^ Besides direct harmful consequences on the individual level, canceled events have significantly impacted numerous aspects of the sports world.

Significant concerns have risen about athletes' safe return to play (RTP) after SARS‐CoV‐2 infection. Training and competing with ongoing myocardial inflammation is not recommended since the sudden cardiac death risk is elevated.^2^ The first RTP recommendations implemented by national sports medicine bodies were based on expert consensus. Finding the optimal RTP examination algorithms and implementing the best policies are of great importance to ensure safety and not to over‐medicalize in uncomplicated cases.

During the first year of the pandemic, study efforts have mainly focused on collegiate athletes. However, the growing popularity of sports among adolescent and master age groups highlights the importance of research in these groups as well.

On top of the acute symptoms, an emerging concern caused by the long COVID syndrome has been described.^3^ In the general population, who have been treated as outpatients, the post‐COVID syndrome may be present in 10%–35% of the cases.^4^ Long COVID syndrome can develop in young, otherwise healthy individuals, even after mildly symptomatic infection,^3^ which might affect the quality and effectiveness of training load build‐up in athletes. Data regarding the prevalence of long COVID syndrome among athletes and its impact on RTP have not been described. Therefore, follow‐up is of the utmost importance to understand the short and mid‐term consequences of the infection.

However, various terms relating to long‐standing symptoms (i.e., long COVID, post‐COVID‐19 syndrome, post‐acute COVID‐19 syndrome, and ongoing symptomatic COVID‐19) are used in scientific literature. We chose the term long COVID to describe subjects who present with persistent or new symptoms beyond four weeks after the acute infection, according to the current NICE guideline.^5^

We set out to conduct a prospective study with two main aims: firstly, to assess disease course, cardiac involvement frequency, and clinical features in various age groups and training levels in athletes with SARS‐CoV‐2 infection, and secondly, to provide detailed information regarding the short and mid‐term consequences of the disease through follow‐up, focusing on the impact and predictors of long COVID and short‐term RTP symptoms in athletes.

## 2 MATERIALS AND METHODS

### 2.1 Study population and recruitment

Between July 2020 and July 2021, we examined 725 athletes median of 24 (IQR 19–32) days after SARS‐CoV‐2 infection at our sports cardiology outpatient clinic.

Competitive athletes presenting for an evaluation after SARS‐CoV‐2 infection had to undergo mandatory cardiac screening involving ECG, echocardiography, and troponin testing per the contemporary regulations implemented by the Hungarian Sports Medicine Institution. On top of the required modalities, we extended our baseline protocol with additional laboratory markers. National sports federations and teams associated with our Institution were the most populous source of subjects. Recreational athletes arrived at their own initiative. We used no active recruitment strategy in this study.

The infection was confirmed by either a rhino pharyngeal swab for SARS‐CoV‐2 RNA (rtPCR), a rapid antigen test, or by the presence of IgG or IgM antibodies at the first examination. Exclusion criteria were as follows: (1) age < 14 years and (2) weekly training volume <3.5 h. None of the examined athletes received a COVID‐19 vaccine before their infection.

Athletes were classified into sports types: skill, power, mixed, and endurance (Table 1).^6^ The athletes were categorized into three symptom severity categories based on the guideline made available by the National Institutes of Health (NIH).^7^ Mild symptoms included fever (body temperature above 38.0 C), sore throat, cough, palpitation (sensation of inappropriately fast heart rate), dizziness, headache, muscle pain, gastrointestinal symptoms (nausea, vomiting, diarrhea), and loss of taste and smell. Athletes presenting with chest pain or dyspnea are categorized as moderately symptomatic.

**TABLE 1:** Baseline demographical, laboratory, imaging and ECG data of the full cohort after SARS‐CoV‐2 infection

	Full sample (*n* = 633)
Age (year, median)	21 (IQR 18–27)
BMI (median)	23 (IQR 22–26)
Sex (Male)	420
Weekly training volume (hours, median)	12 (IQR 8–18)
Elite	15 (IQR 12–20)
Competitive	12 (IQR 8–16)
Recreational	4.5 (IQR 4–6)
National team member	234 (37%)
Hypertension	25 (4%)
Asthma bronchiale	11 (2%)
Known elevated cholesterol levels	24 (4%)
Sports type classification	
Skill	11 (2%)
Power	44 (7%)
Mixed	508 (80%)
Endurance	70 (11%)
Sports type	
Handball	208 (33%)
Basketball	87 (14%)
Waterpolo	83 (13%)
Ice hockey	63 (10%)
Wrestling	30 (5%)
Swimming	27 (4%)
Football (soccer)	23 (4%)
Long‐distance running	18 (3%)
Fencing	7 (1%)
Table tennis	7 (1%)
Kayaking/rowing	6 (1%)
Tennis	5 (1%)
Other	69 (11%)
Symptom severity classification	
Asymptomatic total	102 (16%)
Adolescent	47
Adult	51
Master	4
Mild total	382 (60%)
Adolescent	138
Adult	206
Master	38
Moderate total	149 (24%)
Adolescent	46
Adult	83
Master	20
Symptomatic period (days)	7 (IQR 4–12)
Ferritin above 150 μg/L	95/483 (19.7%)
hsTnT positivity (>14 ng/L)	9/625 (1.4%)
D‐dimer positivity (>0.5 μg/ml)	16/289 (5.5%)
IgM positivity (cutoff = 1 S/Co, *n* = 130)	93 (72%)
IgG positivity (cutoff = 1.4 S/Co, *n* = 458)	397 (86%)
ECG (*n* = 633) alterations	
T‐wave inversion (excluding III, aVR and V1)	12 (2%)
Trifascicular block	1 (<1%)
Echocardiography (*n* = 610)	
Pericardial effusion	7 (1%)
LVEF by echocardiography (%, *n* = 610)	60 (IQR 57–62)
Cardiac magnetic resonance scans (CMR)	104
CMR alterations	7 cases
Pericardial effusion without signs of pericarditis	1 case
Isolated T1 mapping elevation	4 cases
Aspecific, non‐ischaemic LGE	1 case
Possible earlier myocarditis (subepicardial, lateral LGE)	1 case
Computed tomography scans (CT)	24
GGO (ground‐glass opacity)	5 cases
Subpleural atelectasia	1 case
Organising pneumonia	2 cases
Subtle COPD signs	1 case

Abbreviations: CMR, cardiac magnetic resonance; LGE, late gadolinium enhancement; LVEF, left ventricular ejection fraction.

We categorized athletes according to their training load based on the 2020 ESC Guidelines on sports cardiology and exercise.^2^, ^8^ Elites are professional athletes who are members of their national team and generally perform above 10 h per week in training volume. Those who regularly compete and do more than 6 h per week of training but are not national team members are defined as competitive athletes. A third category comprised recreational athletes and referees of a median 4.5 h training volume.

### 2.2 Baseline study protocol

**FIGURE 1:**
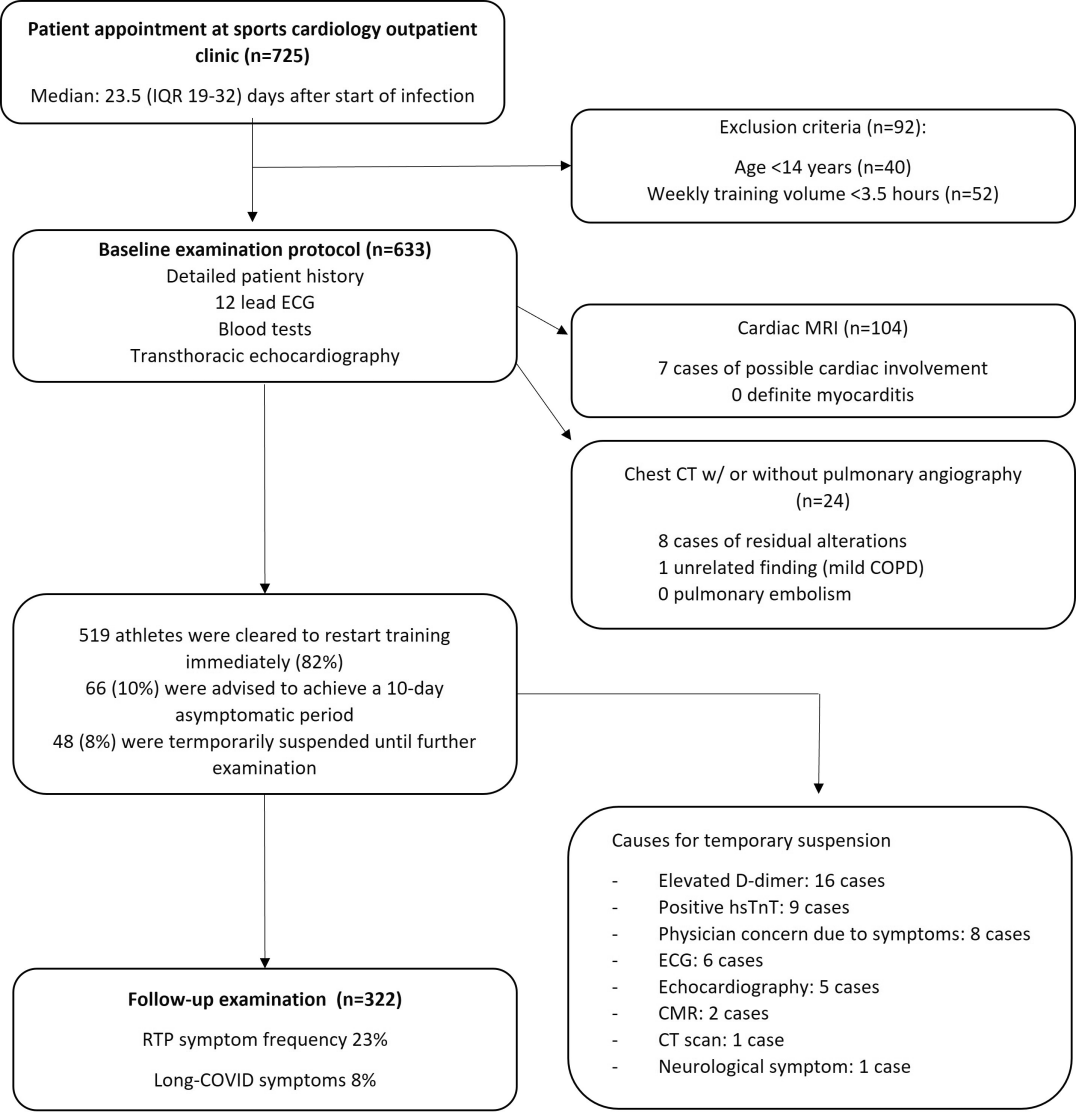
Full examination protocol flowchart. Our final examination group comprised 633 athletes. After carrying out the baseline examination protocol, 82% of the athletes immediately cleared to restart training. Of the 48 (6%) suspended subjects, everyone was able to continue sports activity after the recommended rest period or additional examinations.

Our protocol was based on the Hungarian Sports Medicine Institution's guideline for preparticipation screening protocol, emphasizing cardiac alterations, preferably 2–4 weeks after the commencement of the infection (Figure 1). The first examination took place after a minimum 10‐day‐long quarantine period. It involved physical examination, 12‐lead resting ECG, transthoracic echocardiography, detailed blood tests (including high sensitivity troponin T), and the acquisition of medical history.

ECG was obtained with standard lead positions (MAC 2000, GE Healthcare, Chicago, IL). Transthoracic echocardiography (EPIQ 7G, Philips, Amsterdam, The Netherlands) examinations were carried out at our center, supervised, or carried out by a licensed cardiologist.

### 2.3 Additional examinations

Other examination methods were used based on clinical findings and symptoms, such as the extension of laboratory testing (i.e., D‐dimer) or additional imaging modalities (low‐dose computed tomography scan with or without angiography or CMR). The locally used laboratory kit defined high sensitivity troponin T (hsTnT) cutoff as >13.99 ng/L for positivity, and detection threshold was >2.99 ng/L. D‐dimer was deemed positive above 0.5 μg/ml according to the locally used kit.

Cardiac magnetic resonance (CMR) examinations were carried out on a 1.5 T scanner (Magnetom Aera, Siemens Healthcare, Erlangen, Germany) with a protocol comprising short and long‐axis cine movies, T1 and T2 mapping sequences, and late gadolinium enhancement imaging. Two expert readers evaluated the images.

We defined possible peri/myocardial involvement if (1) slightly elevated T1 mapping value or late gadolinium enhancement (LGE) with normal T2 times or (2) slightly elevated T1 and T2 mapping values were present with no LGE. We used the updated Lake Louise criteria to diagnose definite myocarditis, if any.

### 2.4 Follow‐up

Subjects were offered a follow‐up examination within 1–6 months of their acute infection. The follow‐up examination involved detailed blood testing and an ECG. We assessed long COVID symptoms by a standardized questionnaire regarding the circumstances of resumption of training (i.e., presence of long‐standing symptoms, time taken to rebuild training load) and asked for feedback about the examination process.

Athletes with a shorter symptomatic period associated with exertion of maximum two weeks after restarting to train (minimal mandatory quarantine period was ten days) were regarded as subjects with short‐term RTP symptoms.

The long COVID group comprised individuals presenting with acute symptoms lasting for more than four weeks.^5^, ^9^, ^10^ All symptoms that might have affected athletic performance were included (i.e., loss of smell or taste as the only long‐standing sign did not qualify as long COVID in this case).

There was no overlap between the two categories.

### 2.5 Data management, statistical analysis

We used the Shapiro–Wilk to test data normality. Continuous variables are presented as mean ± SD or median and interquartile range as appropriate. Comparison between two independent groups was performed by t‐tests or Mann–Whitney test. In the analyses involving three different groups, the Kruskal–Wallis test was used. *p*‐Values <0.05 were considered significant. Chi‐squared tests were applied to compare the distributions of categorical data.

We used the LightGBM gradient boosting framework to build a logistic regression model. A tree‐based learning algorithm identified predictors and laboratory cutoffs used in the analysis. Missing continuous variables were complemented with the average where it applied in model building.

Statistical analyses were conducted by MedCalc's software v20.013 (MedCalc Software Ltd, Ostend, Belgium) and Python 3.9.

## 3 RESULTS

### 3.1 Baseline characteristics

The final study cohort consisted of 633 competitive and recreational athletes (male = 420) after exclusions. Adolescents were 14‐ to 19‐year‐old, adults were between 20 and 35 years old, and subjects above 35 years were regarded as master athletes. Eighty‐nine percent (*n* = 563/633) of the included athletes compete at least on a national level. Thirty‐seven percent of the cohort (*n* = 234/633) were members of their national team (elite athletes). The remaining portion of our cohort comprised recreational athletes and referees. Handball players were the most dominant in the cohort, with 33%.

We performed the sports cardiology assessment median 24 (IQR 19–32) days after the acute SARS‐CoV‐2 infection. Asymptomatic infection occurred in 16% of the cases; 60% were mildly symptomatic, and the remaining 24% presented moderate symptoms.

During the baseline evaluation, high sensitivity troponin T (hsTnT) and D‐dimer positivity were rare (Table 1).

ECG and echocardiography alterations that indicated further examination were rare (Figure 1). As a possible sign of myocardial involvement, T‐wave inversion was present in 2% of the athletes. Pericardial effusion was found in only 7 cases (1%). Of the CMR 104 scans, 97% were performed using a gadolinium‐based contrast agent, and 3 were native scans where patients did not consent to receive contrast agent. We found alterations suggestive of possible peri/myocardial involvement in only 7 cases (7%). There was no evidence of ongoing, definite myocardial inflammation per the updated Lake Louise Criteria.^11^ In 59% (*n* = 61) of the cases, the physician's concern indicated the CMR. Six scans were indicated due to elevated troponin levels, 13 due to ECG alterations, 15 due to echocardiography abnormalities, eight due to prolonged chest complaints, and one due to previously diagnosed pericarditis.

### 3.2 Return to play (RTP) and follow‐up

The majority (82%) of the athletes were cleared to RTP without further investigation. Only 8% (*n* = 48) of the athletes had to be temporarily restricted from training resumption due to initial abnormal findings. The remaining 10% were advised to achieve a 10‐day asymptomatic period before restarting to train (Figure 1). Athletes who completed a 10‐day asymptomatic period and had no alteration suggesting myocardial involvement were advised to gradually build up training load with heart rate control. To those who sought our counsel during the acute period of the infection, we recommended rest or low‐intensity resistance training in case of being asymptomatic or mildly symptomatic. To provide RTP guidance after recovery from the disease, the participating sports medicine experts at our Institution recommended a target heart rate of approximately 60% (based on the 220‐age formula) on the first week, 80% on the second week of RTP and continuation toward maximum training load from the third week.

A small number of athletes was primarily examined because of long‐standing symptoms (*n* = 18).

**FIGURE 2:**
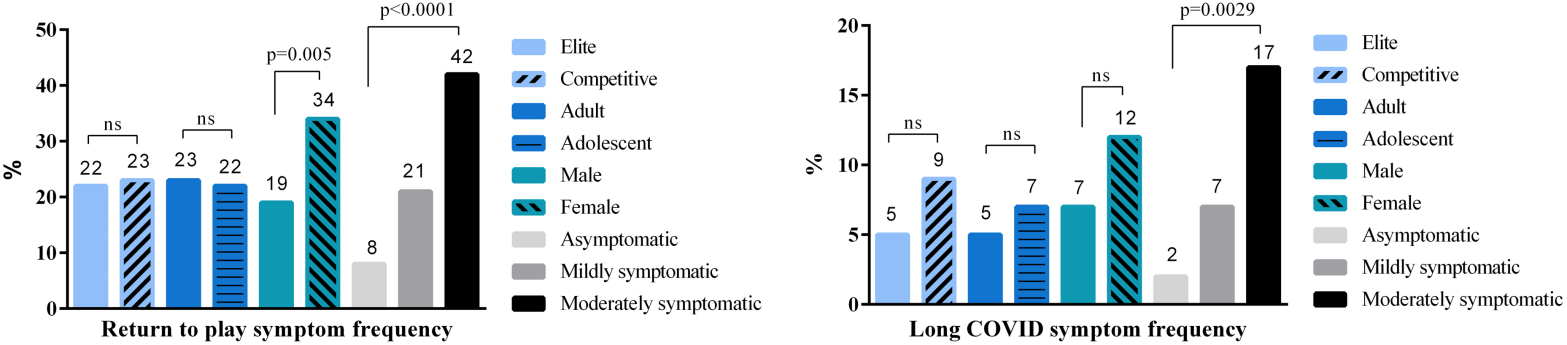
Distribution of return to play (RTP) and long COVID symptom frequency among different athlete groups after follow‐up. There was a significant difference between male and female subjects in RTP symptom frequency and between symptom severity categories. Long COVID symptom frequency showed similar significant differences in symptom severity classification.

A follow‐up examination was conducted in 322 cases (median 107 days after the start of the infection). We investigated the burden of short‐term RTP symptoms, signaling reoccurring or prolonged symptoms while restarting to train, and long COVID symptoms persisting over four weeks. Athletes reported RTP symptoms at a frequency of 23% (*n* = 74), on top 8% (*n* = 28) had long COVID symptoms. The most common short‐term RTP symptoms were prolonged fatigue (45%) and palpitations (39%). Short‐term RTP symptoms were more frequent (34% vs. 19%, *p* = 0.005) in female athletes than in male athletes (Figure 2). However, female athletes reported needing slightly less time to rebuild maximal training intensity and peak form (median 3 vs. 4 weeks, *p* = 0.01). There was a significant difference in long COVID symptom frequency between different age groups and symptom severity groups, whereas short‐term RTP symptoms have only shown a difference regarding symptom severity (Table 2). Antibody levels (IgG) have shown a significant decrease within the follow‐up period but remained above the cutoff (1.4 S/Co) determined by the manufacturer (median 3.9 vs. 2.0 S/Co, *p* < 0.0001).

**TABLE 2:** Comparison of the different symptomatic and age groups

	Asymptomatic SARS‐CoV‐2 infection (*n* = 102)	Mild symptoms (*n* = 382)	Moderate symptoms (*n* = 149)	*p* Value	Adolescents (*n* = 231)	Adults (*n* = 330)	Masters (*n* = 62)	*p* Value
Age (IQR)	20 (18–24)	22 (17–28)	23 (19–29)	<0.05*,**	17 (15–18)	24 (21–28)	43 (37–47)	<0.05^#^
BMI (IQR)	23 (22–25)	24 (22–26)	23 (22–25)	NS	22 (21–24)	24 (22–26)	26 (23–28)	<0.0001^#^
National team member	49	126	60	0.0133^#^	83	143	9	0.0002^#^
Weekly training volume (hours, IQR)	14 (8–20)	12 (8–17)	12 (8–16)	NS	13 (9–20)	13 (9–18)	6 (4.5–9)	<0.05^††^,^†††^
Symptomatic days (IQR)	0	6 (3–10)	9 (5–16)	<0.0001***	7 (4–12)	7 (4–10)	9 (4–18)	NS
RTP symptom frequency (*n* = 74/322)	4/55 (7%)	41/196 (21%)	29/69 (42%)	<0.0001^#^	31/143 (22%)	38/162 (31%)	5/15 (33%)	NS
Long COVID symptom frequency (*n* = 28/322)	1/59 (2%)	14/200 (7%)	13/73 (17%)	0.02**	7/145 (5%)	12/171 (7%)	9/22 (41%)	<0.0001^#^
Time to max. Training intensity (weeks, IQR)	3 (1–5)	4 (2–5)	4 (2–6)	NS	4 (2–5)	4 (2–5)	4 (2–8)	NS
Time to peak form (weeks, IQR)	5 (2–6)	5 (4–7)	6 (4–8)	NS	5 (3–7)	6 (4–8)	6 (4–8)	NS

*Note*: The chi‐squared test or Kruskal‐Wallis test was used to compare the groups as appropriate.

Abbreviations: BMI, body‐mass index; IQR, interquartile range; NS, non‐significant; RTP, return‐to‐play.

* Significant difference between asymptomatic and mildly symptomatic subjects.

** Significant difference between asymptomatic and moderately symptomatic subjects.

*** Significant difference between mildly and moderately symptomatic subjects.

^††^ Significant difference between Adolescents and Masters.

^†††^ Significant difference between Adults and Masters.

^#^ Significant difference between three individual groups.

Ferritin levels have decreased, albeit remained in the normal range at baseline and follow‐up examinations (median 78 vs. 68 μg/L, *p* < 0.0001). Athletes reported a 7.5% frequency of novel sports injuries after training resumption, which did not show an association with training pause length (*p* = 0.94). Detailed baseline and follow‐up laboratory findings are presented in Table 3.

**TABLE 3:** Detailed laboratory parameters of the cohort after the baseline and follow‐up examinations

	Baseline value—Median (IQR)	Number of samples	Follow‐up value—Median (IQR)	Number of samples
High sensitivity troponin T (ng/L)	4 (3–6)	616	6 (4–8)	204
D‐dimer^a^	0.3 (0.3–0.3)	288	0.3 (0.3–0.3)	13
Ferritin (μg/L)	71 (39–127)	483	73 (44–118)	222
25OH Vitamin D (ng/ml)	33 (26–43)	459	30 (24–38)	219
CRP (mg/L)	0.6 (0.3–1.1)	611	0.7 (0.6–1.4)	201
GOT (U/L)	20 (17–24)	597	24 (20–28)	199
GPT (U/L)	16 (12–23)	606	17 (23–24)	205
GGT (U/L)	13 (10–18)	606	13 (10–16)	202
ALP (U/L)	72 (57–91)	606	81 (62–104)	200
Creatinine (μmol/L)	77 (68–87)	604	81 (72–92)	199
Haemoglobin (g/L)	150 (140–159)	614	151 (141–158)	209
WBC count (G/L)	6.1 (5.2–7.1)	614	5.8 (4.9–6.9)	208
Neutrophil granulotyte count (G/L)	3.3 (2.6–4.1)	601	3.0 (2.4–4.1)	207
Lymphocyte count (G/L)	2.0 (1.6–2.4)	601	1.9 (1.6–2.2)	207
IgM (S/Co)	1.7 (0.9–4.0)	130	0.6 (0.3–1.2)	99
IgG (S/Co)	3.9 (2.2–5.6)	458	1.8 (0.9–3.1)	246

*Note*: Out‐of‐range data is found in Table 1.

Abbreviations: ALP, Alkaline phosphatase; CRP, C‐reactive protein; GGT, gamma‐ glutamyl Transferase; GOT, glutamic‐oxaloacetic transaminase; GPT, glutamic‐pyruvic transaminase.

^a^ D‐dimer's detection threshold was 0.3 with the locally used laboratory kit.

### 3.3 The predictors of persistent symptoms

**FIGURE 3:**
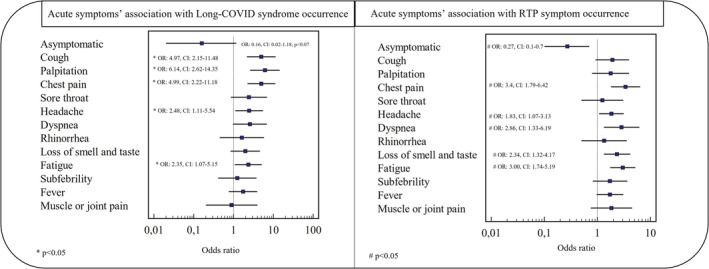
Forest plot showing the association between different acute symptoms and subsequent RTP or long COVID symptom development. In terms of Long COVID syndrome occurrence, cough, palpitation and chest pain carried the highest odds ratios. RTP symptoms showed the strongest association with chest pain, dyspnea and fatigue. An asymptomatic infection had a significantly lower odds ratio in the RTP symptom analysis. A similar tendency was observable in long COVID syndrome occurrence. OR, odds ratio; RTP, return to play.

Some specific acute symptoms have shown an individual association with developing long COVID and return to play symptoms (Figure 3). Asymptomatic infection implied a lower chance of developing short‐term RTP symptoms (OR 0.27, CI 0.1–0.7) and a similar but non‐significant tendency for long COVID symptoms (OR 0.16, CI 0.02–1.18).

Moderately symptomatic individuals have shown more disposition to develop long COVID symptoms than mildly symptomatic ones (7% vs. 17%, *p* = 0.02). In the same way, short‐term RTP complaint frequency was also associated with more severe acute symptoms (*p* < 0.0001).

Athletes presenting with either or both cough and ferritin levels higher than >150 μg/L carried a 4.1x (odds ratio CI 1.78–9.6, *p* = 0.001) higher risk of developing long COVID symptoms (21% vs. 6%).

Based on biological plausibility, we selected age, symptom severity, and baseline ferritin levels in a multivariate logistic regression model to predict long COVID occurrence. In our model, increasing age and higher symptom severity scores were independent predictors of long COVID (AUC 0.746, CI 0.685–0.801). Similarly, we included age, sex, weekly training volume, baseline ferritin levels, and symptom severity scores in predicting short‐term RTP symptoms. Sex and symptom severity scores were independent predictors in the model (AUC 0.72, CI 0.655–0.779).

In addition, a small proportion (*n* = 40/250, 16%) of athletes who had given feedback on this matter did not pause training while being infected against medical advice, albeit it did not seem to increase short‐term RTP symptom frequency (*p* = 0.44).

## 4 DISCUSSION

We assessed a high‐volume athletes' cohort's outcomes after SARS‐CoV‐2 infection, which is currently the largest one published in the European region. None of the subjects was vaccinated against SARS‐CoV‐2 before contracting the virus. Cardiac or possible cardiac involvement was proven to be expressly low in the examined athlete group(s).

The first significant article regarding COVID‐19's myocardial effects investigated by CMR reported an alarmingly high prevalence of myocardial damage in recovered COVID‐19 patients.^12^ In another CMR study by Rajpal et al., 15% of the examined athletes (*n* = 26) fulfilled the two main features of the updated Lake Louise criteria suggestive of myocarditis.^13^ Brito et al. reported pericardial inflammation in more than 1 in 3 previously healthy college athletes, where 96% of the examined subjects showed mild or no symptoms.^14^ These results kept concerns high for athletes going through COVID‐19 infection and facilitated extensive testing in this population.

Moulson et al. have recently published the findings of 3018 athletes suggesting that asymptomatic or mildly symptomatic athletes may return to sport without cardiac testing, preserving 12‐lead ECG and transthoracic echocardiography for moderately symptomatic individuals. In addition, they concluded that CMR is the most useful in athletes with high pretest probability.^15^ Cavigli et al. have recently reported their experiences with junior competitive athletes wherein a cohort of 571 subjects found that half of the athletes had had only mild symptoms and pericardial involvement was rare, examined by transthoracic echocardiography. They concluded that echocardiography should be reserved for those with cardiopulmonary symptoms or ECG abnormalities.^16^

Initial findings at our center suggested no evident signs of myocardial involvement on CMR examination in twelve elite athletes who recovered from COVID‐19.^17^ By broadening our research to 147 subjects, definite signs of myocarditis were as scarcely prevalent as 1.4% in the whole cohort.^18^ The subjects who underwent CMR presented in this work partially overlap with our comprehensive CMR study cohort.

Also, a multicenter cross‐sectional publication by Martinez et al. has shown a low prevalence of myocardial involvement in professional athletes in a high‐volume study from North American centers. However, their sample was almost an all‐male one with a 98.5% proportion. Only 0.6% of the athletes had to be restricted from training resumption due to potential inflammatory heart disease.^19^

Fikenzer et al. proposed performing spiroergometry to guide individual athletes' return better to play plan after excluding peri‐ or myocardial involvement with echocardiography or CMR.^20^ While this might be useful to aid in rebuilding training load, it should be considered that asymptomatic and mildly symptomatic athletes' screening with extensive cardiac imaging and spiroergometry may not add relevant information in uncomplicated cases but acts as an extra burden on sports cardiology care providers, as it was concluded by other authors as well.^21^

Top‐level athletes are primarily young adults who have been less likely to need inpatient care with COVID‐19 infection and do not carry typical risk factors for developing a severe disease course.^22^ In addition, muscle strength seems to be a protective factor against hospitalization related to COVID‐19 in middle‐aged and older adults.^23^ Of the 728 athletes presenting at our outpatient clinic, only 0.4% (*n* = 3) were hospitalized due to COVID. In a study conducted in the United States, 5% of 63 103 young adults coded as COVID‐19 patients aged between 18 and 34 needed hospitalization with COVID‐19.^24^ In a Spanish cohort of 420 individuals, self‐reported moderate or vigorous physical activity patterns were inversely associated with hospitalization for respiratory symptoms.^25^ Brawner et al.'s findings suggest that maximal exercise capacity is inversely related to the likelihood of hospitalization due to SARS‐CoV‐2.^26^ These results indicate that regular physical activity may have a protective role in the case of COVID‐19, similarly to numerous other diseases.^27^

Additional factors such as skeletal muscle mass, body fat percentage, and body composition might also play a role in forming the disease course of COVID‐19. Perez‐Valera, Martinez‐Canton et al. proved that ACE2 (the receptor of SARS‐CoV‐2) expression in the skeletal muscle is more prominent in those with higher percentages of body fat. As athletes generally bear higher lean muscle mass and lower body fat percentage than their non‐athlete counterparts, ACE2 expression pattern in the skeletal muscle might also be a modifying factor in the disease course of COVID‐19.^28^ This is also underlined by a histopathology‐based study involving sixteen patients' muscle biopsies with persistent fatigue, weakness, or myalgia. A range of histological changes, including mitochondrial damage and inflammation, have been confirmed, making skeletal muscle a primary target for SARS‐CoV‐2.^29^

Moreno‐Pérez et al. reported that post‐acute COVID‐19 syndrome might be present in up to 50% of COVID‐19 survivors in a median 56‐year‐old cohort.^30^ Sudre et al. carried out a study in which participants self‐reported post‐COVID symptoms through a mobile phone application, where 13.3% of the subject reported symptoms lasting longer than 28 days. They found an association between long COVID and the number of symptoms in the first week, increasing age, female sex, and BMI.^31^ Our athletes showed a lower prevalence of long COVID symptoms than the aforementioned non‐selected sample. Nevertheless, Petek et al. found even lower persistent and exertional symptom occurrence (5.4%) in an observational cohort of 3597 collegiate athletes.^32^

Our findings suggest that specific acute symptoms might be able to predict worse disease courses in a cohort of mainly young and adult athletic individuals with reasonable accuracy. These athletes at risk of developing persisting or reoccurring symptoms may benefit from individual assessment and careful planning of training load rebuild. Ferritin levels remained in the normal range for the vast majority of the athletes upon baseline and follow‐up examination. In whom it was above 150 μg/L at the baseline examination carried a higher risk of developing long COVID symptoms. At the same time, these values did not come near the ferritin levels seen in severely ill patients.^33^

Master athletes with more severe symptoms were more likely to develop long COVID syndrome reported similar time needed to achieve maximal training intensity as younger groups. This might be because master athletes perform sports at a lower level, and the proportion of them being competitive athletes is also lower than younger athletes. Kim et al. proposed a screening algorithm for master athletes based on symptom severity, including considering a CMR in moderately symptomatic cases with an abnormal finding in either of the baseline methods. They also emphasized the importance of shared decision‐making in case of uncertain findings (not only in master athletes), similar to other cardiac pathologies concerning athletes.^34^, ^35^

## 5 PERSPECTIVE

In a cohort of 633 adolescent, adult, and master athletes, we found expressly low myocardial involvement after SARS‐CoV‐2 infection. Persistent and reoccurring symptoms may pose a problem in keeping and rebuilding fitness levels, especially during the mid‐season. Long COVID and RTP symptoms hindered rapid training load and form rebuild in a significant portion of athletes, as 31% reported prolonged COVID‐related health issues. Specific acute symptoms (chest pain, cough, dyspnea) and ferritin levels may be associated with short and mid‐term outcomes. An individualized approach based on acute symptom severity and baseline laboratory (if indicated) findings is favorable in determining when to clear athletes for return to play. Clinicians and trainers could better guide return‐to‐play timing and training load rebuild pace by identifying athletes with a predisposition to developing long‐standing symptoms.

## 6 STRENGTHS AND LIMITATIONS

We present a single‐center study conducted by a major regional cardiac institution. One strength of our research is the homogenous laboratory and imaging testing methods. Best to our knowledge, this is the largest single‐center post‐COVID athletes' research up to date.

A portion of subjects presented for a post‐COVID screening more than four weeks after the commencement of their infection; this might have reduced the frequency of identifiable subclinical abnormalities. Given the relatively small number of master athletes in this study, more data are required to accurately assess their outcomes and the extent of their possible protection against developing a severe disease course. Also, the definition and categorization of long COVID were based on self‐reported symptoms as we lacked objective measurements and descriptions. However, this was unavoidable in our current state of knowledge during the study period.

Almost half of the subjects did not return for a follow‐up examination. The reasons might be that the first baseline visit was mandatory for athletes to regain their license to compete but not a follow‐up, especially within a shorter time interval than usual (a significant proportion of our athletes present at our clinic once a year). Also, athletes with long‐standing symptoms might have been more prone to seek medical attention, which may stand for possible selection bias.

Referral for a CMR examination involved a selected group of athletes based on symptom severity or abnormal baseline examination findings. By this means, our relatively favorable CMR findings may still overestimate possible myocardial involvement in athletes.

## 7 FEEDBACK FROM THE ATHLETES

Two hundred thirty‐three athletes agreed to fill in a feedback questionnaire regarding the examination workflow. Seventy‐five percent of the athletes (*n* = 175) reported that the examination had provided an extra sense of security for them before the resumption of training 98% of them were overall satisfied with the process. According to the athletes' answers, the average time needed to complete the first examination protocol took, on average, 92 min. More than half of the subjects (53%) looked up information on COVID‐19's implications on athletes' health before their appointment. Their primary sources were the Internet (34%), their team doctor(s) (25%), team crew or sports federation (18%), and news or television (7%).

## AUTHOR CONTRIBUTIONS

VJ contributed to the conception of the study, investigation, analysis, project administration, interpretation of the data, and writing the original draft. LS contributed to the conception of formal data analysis, investigation, analysis, and editing of the manuscript. AT and AP contributed to project organization, conceptualization, and investigation. OK, MB, NS, DB, ZG, and EC contributed to the formal analysis of the data, investigation, and project administration. AB and AO contributed to the advanced analysis of the data. DB and BM contributed to the conceptualization, interpretation of the data, funding acquisition, supervision, and manuscript editing. HV contributed to the conceptualization, investigation, interpretation of the data, funding acquisition, supervision, writing, and manuscript editing. All authors gave final approval to the manuscript.

## FUNDING INFORMATION

Project no. NVKP_16‐1–2016‐0017 (“National Heart Program”) has been implemented with the support provided by the National Research, Development, and Innovation Fund of Hungary, financed under the NVKP_16 funding scheme. The research was financed by the Thematic Excellence Programme (2020‐4.1.1.‐TKP2020) of the Ministry for Innovation and Technology in Hungary, within the framework of the Therapeutic Development and Bioimaging thematic programs of Semmelweis University. The research presented in this paper, was supported by the European Union project RRF‐2.3.1‐21‐2022‐00004 within the framework of the Artificial Intelligence National Laboratory. This project was also supported by a grant from the National Research, Development, and Innovation Office (NKFIH) of Hungary (K135076 to BM). This project was supported by a grant from the National Research, Development, and Innovation Office (NKFIH) of Hungary (2020‐1.1.6‐JÖVŐ‐2021‐00013).

## CONFLICT OF INTEREST

The authors declare no conflicts of interest.

## ETHICS APPROVAL AND CONSENT TO PARTICIPATE

Ethical approval was obtained from the National Public Health Centre per the ethical standards laid out in the 1964 Declaration of Helsinki and its later amendments (IV/9697‐1/2020/EKU). All participants or their guardians gave their written informed consent.

## Data Availability

The data supporting this study's findings are available at reasonable request from the corresponding author.

## References

[1] Johns Hopkins University & Medicine . 2022. https://coronavirus.jhu.edu/map.html. Accessed March 10, 2022.

[2] Pelliccia A , Sharma S , Gati S , et al. 2020 ESC Guidelines on sports cardiology and exercise in patients with cardiovascular disease. Eur Heart J. 2021;42(1):17‐96. 33180902 10.1093/eurheartj/ehaa735

[3] Crook H , Raza S , Nowell J , Young M , Edison P . Long covid‐mechanisms, risk factors, and management. BMJ [Internet]. 2021;374:n1648. http://www.ncbi.nlm.nih.gov/pubmed/34312178. Accessed October 12, 2021. 34312178 10.1136/bmj.n1648

[4] Pavli A , Theodoridou M , Maltezou HC . Post‐COVID syndrome: incidence, clinical spectrum, and challenges for primary healthcare professionals. Arch Med Res. 2021;52:575‐581. 33962805 10.1016/j.arcmed.2021.03.010 PMC8093949

[5] National Institute for Health and Care Excellence (NICE) SIGN (SIGN) and RC of GP (RCGP) . 2022. https://www.nice.org.uk/guidance/ng188/resources/covid19‐rapid‐guideline‐managing‐the‐longterm‐effects‐of‐covid19‐pdf‐51035515742. Accessed January 20, 2022.

[6] Niebauer J , Börjesson M , Carre F , et al. Recommendations for participation in competitive sports of athletes with arterial hypertension: a position statement from the sports cardiology section of the European Association of Preventive Cardiology (EAPC). Eur Heart J. 2018;39(40):3664‐3671. 30165596 10.1093/eurheartj/ehy511

[7] NIH . 2020. Clinical Spectrum of SARS‐CoV‐2 Infection [Internet]. https://www.covid19treatmentguidelines.nih.gov/overview/clinical‐spectrum/. Accessed October 12, 2021.

[8] McKinney J , Velghe J , Fee J , Isserow S , Drezner JA . Defining athletes and exercisers. Am J Cardiol. 2019;123:532‐535. 30503799 10.1016/j.amjcard.2018.11.001

[9] Nalbandian A , Sehgal K , Gupta A , et al. Post‐acute COVID‐19 syndrome. Nat Med. 2021;27:601‐615. 33753937 10.1038/s41591-021-01283-z PMC8893149

[10] Datta SD , Talwar A , Lee JT . A proposed framework and timeline of the spectrum of disease due to SARS‐CoV‐2 infection: illness beyond acute infection and public health implications. JAMA. 2020;324:2251‐2252. 33206133 10.1001/jama.2020.22717

[11] Ferreira VM , Schulz‐Menger J , Holmvang G , et al. Cardiovascular magnetic resonance in nonischemic myocardial inflammation: expert recommendations. J Am Coll Cardiol. 2018;72(24):3158‐3176. 30545455 10.1016/j.jacc.2018.09.072

[12] Puntmann VO , Carerj ML , Wieters I , et al. Outcomes of cardiovascular magnetic resonance imaging in patients recently recovered from coronavirus disease 2019 (COVID‐19). JAMA Cardiol. 2020;5(11):1265‐1273. 32730619 10.1001/jamacardio.2020.3557 PMC7385689

[13] Rajpal S , Tong MS , Borchers J , et al. Cardiovascular magnetic resonance findings in competitive athletes recovering from COVID‐19 infection. JAMA Cardiol. 2021;6(1):116‐118. 32915194 10.1001/jamacardio.2020.4916 PMC7489396

[14] Brito D , Meester S , Yanamala N , et al. High prevalence of pericardial involvement in college student athletes recovering from COVID‐19. JACC Cardiovasc Imaging. 2020;14(3):541‐555. 33223496 10.1016/j.jcmg.2020.10.023 PMC7641597

[15] Moulson N , Petek BJ , Drezner JA , et al. SARS‐CoV‐2 cardiac involvement in young competitive athletes. Circulation [Internet]. 2021;144:256‐266. doi:10.1161/CIRCULATIONAHA.121.054824 33866822 PMC8300154

[16] Cavigli L , Cillis M , Mochi V , et al. SARS‐CoV‐2 infection and return to play in junior competitive athletes: is systematic cardiac screening needed? Br J Sports Med. 2021;56(5):264‐270. doi:10.1136/bjsports-2021-104764 34844952

[17] Vago H , Szabo L , Dohy Z , Merkely B . Cardiac magnetic resonance findings in patients recovered from COVID‐19: initial experiences in elite athletes. JACC Cardiovasc Imaging. 2021;14(6):1279‐1281. 33341416 10.1016/j.jcmg.2020.11.014 PMC7837171

[18] Szabó L , Juhász V , Dohy Z , et al. Is cardiac involvement prevalent in highly trained athletes after SARS‐CoV‐2 infection? A cardiac magnetic resonance study using sex‐matched and age‐matched controls. Br J Sports Med. 2021;56(10):553‐560. doi:10.1136/bjsports-2021-104576 34848398 PMC8637606

[19] Martinez MW , Tucker AM , Bloom OJ , et al. Prevalence of inflammatory heart disease among professional athletes with prior COVID‐19 infection who received systematic return‐to‐play cardiac screening. JAMA. 2021;10019:1‐8. 10.1001/jamacardio.2021.0565 PMC7934073 33662103

[20] Fikenzer S , Kogel A , Pietsch C , et al. SARS‐CoV2 infection: functional and morphological cardiopulmonary changes in elite handball players. Sci Rep. 2021;11(1):17798 https://www.nature.com/articles/s41598‐021‐97120‐x. Accessed December 6, 2021. 34493765 10.1038/s41598-021-97120-x PMC8423785

[21] Gervasi SF , Pengue L , Damato L , et al. Is extensive cardiopulmonary screening useful in athletes with previous asymptomatic or mild SARS‐CoV‐2 infection? Br J Sports Med. 2021;55(1):54‐61. 33020140 10.1136/bjsports-2020-102789 PMC7536638

[22] Richardson S , Hirsch JS , Narasimhan M , et al. Presenting characteristics, comorbidities, and outcomes among 5700 patients hospitalized with COVID‐19 in the New York City area. JAMA. 2020;323(20):2052‐2059. 32320003 10.1001/jama.2020.6775 PMC7177629

[23] Maltagliati S , Sieber S , Sarrazin P , et al. Muscle strength explains the protective effect of physical activity against COVID‐19 hospitalization among adults aged 50 years and older. J Sports Sci. 2021;39(24):2796‐2803. doi:10.1080/02640414.2021.1964721 34376100

[24] Cunningham JW , Vaduganathan M , Claggett BL , et al. Clinical outcomes in young US adults hospitalized with COVID‐19. JAMA Intern Med. 2021;181:379‐381. 10.1001/jamainternmed.2020.5313 PMC7489373 32902580

[25] Latorre‐Román PÁ , Guzmán‐Guzmán IP , Delgado‐Floody P , et al. Protective role of physical activity patterns prior to COVID‐19 confinement with the severity/duration of respiratory pathologies consistent with COVID‐19 symptoms in Spanish populations. Res Sports Med. 2021;1‐12. doi:10.1080/15438627.2021.1937166 34128446

[26] Brawner CA , Ehrman JK , Bole S , et al. Inverse relationship of maximal exercise capacity to hospitalization secondary to coronavirus disease 2019. Mayo Clin Proc. 2021;96(1):32‐39. 33413833 10.1016/j.mayocp.2020.10.003 PMC7547590

[27] Kasiakogias A , Sharma S . Exercise: the ultimate treatment to all ailments? Clin Cardiol. 2020;43:817‐826. 32506511 10.1002/clc.23369 PMC7403692

[28] Perez‐Valera M , Martinez‐Canton M , Gallego‐Selles A , et al. Angiotensin‐converting enzyme 2 (SARS‐CoV‐2 receptor) expression in human skeletal muscle. Scand J Med Sci Sports. 2021;31(12):2249‐2258. 34551157 10.1111/sms.14061 PMC8662278

[29] Hejbøl EK , Harbo T , Agergaard J , et al. Myopathy as a cause of fatigue in long‐term post‐COVID‐19 symptoms: evidence of skeletal muscle histopathology. Eur J Neurol. 2022;29(9):2832‐2841. 35661354 10.1111/ene.15435 PMC9348124

[30] Moreno‐Pérez O , Merino E , Leon‐Ramirez JM , et al. Post‐acute COVID‐19 syndrome. Incidence and risk factors: a Mediterranean cohort study. J Infect. 2021;82(3):378‐383. 33450302 10.1016/j.jinf.2021.01.004 PMC7802523

[31] Sudre CH , Murray B , Varsavsky T , et al. Attributes and predictors of long COVID. Nat Med. 2021;27(4):626‐631. 33692530 10.1038/s41591-021-01292-y PMC7611399

[32] Petek BJ , Moulson N , Baggish AL , et al. Prevalence and clinical implications of persistent or exertional cardiopulmonary symptoms following SARS‐CoV‐2 infection in 3597 collegiate athletes: a study from the Outcomes Registry for Cardiac Conditions in Athletes (ORCCA). Br J Sports Med. 2021;56(16):913‐918. http://www.ncbi.nlm.nih.gov/pubmed/34725052. Accessed February 2, 2022. 34725052 10.1136/bjsports-2021-104644 PMC8561826

[33] Gómez‐Pastora J , Weigand M , Kim J , et al. Hyperferritinemia in critically ill COVID‐19 patients—is ferritin the product of inflammation or a pathogenic mediator? Clin Chim Acta. 2020;509:249‐251. 32579952 10.1016/j.cca.2020.06.033 PMC7306200

[34] Kim JH , Levine BD , Phelan D , et al. Coronavirus disease 2019 and the athletic heart: emerging perspectives on pathology, risks, and return to play. JAMA Cardiol. 2020;6(2):219‐227. 10.1001/jamacardio.2020.5890 33104154

[35] Baggish AL , Ackerman MJ , Lampert R . Competitive sport participation among athletes with heart disease: a call for a paradigm shift in decision making. Circulation. 2017;136(17):1569‐1571. 29061571 10.1161/CIRCULATIONAHA.117.029639

